# Preoperative Prediction of Central Cervical Lymph Node Metastasis in Fine-Needle Aspiration Reporting Suspicious Papillary Thyroid Cancer or Papillary Thyroid Cancer Without Lateral Neck Metastasis

**DOI:** 10.3389/fonc.2022.712723

**Published:** 2022-03-23

**Authors:** Kai Zhang, Lang Qian, Jieying Chen, Qian Zhu, Cai Chang

**Affiliations:** ^1^ Department of Medical Ultrasound, Fudan University Shanghai Cancer Center, Shanghai, China; ^2^ Department of Oncology, Shanghai Medical College of Fudan University, Shanghai, China; ^3^ Department of Radiology, Shanghai Tenth People’s Hospital, Tongji University School of Medicine, Shanghai, China

**Keywords:** papillary thyroid cancer, thyroid cancer, cervical lymph node, lymph node metastasis, central neck dissection

## Abstract

**Purpose:**

No non-invasive method can accurately determine the presence of central cervical lymph node (CCLN) metastasis in papillary thyroid cancer (PTC) until now. This study aimed to investigate factors significantly associated with CCLN metastasis and then develop a model to preoperatively predict CCLN metastasis in fine-needle aspiration (FNA) reporting suspicious papillary thyroid cancer (PTC) or PTC without lateral neck metastasis.

**Patients and Methods:**

Consecutive inpatients who were diagnosed as suspicious PTC or PTC in FNA and underwent partial or total thyroidectomy and CCLN dissection between May 1^st^, 2016 and June 30^th^, 2018 were included. The total eligible patients were randomly divided into a training set and an internal validation set with the ratio of 7:3. Univariate analysis and multivariate analysis were conducted in the training set to investigate factors associated with CCLN metastasis. The predicting model was built with factors significantly correlated with CCLN metastasis and validated in the validation set.

**Results:**

A total of 770 patients were eligible in this study. Among them, 268 patients had histologically confirmed CCLN metastasis, while the remaining patients did not. Factors including age, *BRAF* mutation, multifocality, size, and capsule involvement were found to be significantly correlated with the CCLN metastasis in univariate and multivariate analysis. A model used to predict the presence CCLN metastasis based on these factors and US CCLN status yielded AUC, sensitivity, specificity and accuracy of 0.933 (95%CI: 0.905-0.960, p < 0.001), 0.816, 0.966 and 0.914 in the training set and 0.967 (95%CI: 0.943-0.991, p < 0.001), 0.897, 0.959 and 0.936 in the internal validation set.

**Conclusion:**

Age, *BRAF* mutation, multifocality, size, and capsule involvement were independent predictors of CCLN metastasis in FNA reporting suspicious PTC or PTC without lateral neck metastasis. A simple model was successfully built and showed excellent discrimination to distinguish patients with or without CCLN metastasis.

## Introduction

Thyroid cancer ranks the seventh in the list of newly diagnosed cancers and occurs 3.2 times more often in women than in men in China according to the latest report (1). The mortality increased slightly from 2003 to 2012, whereas the incidence increased by approximately five times during the same period (1), indicating more medical resource is needed to invest in treating this disease. Papillary thyroid cancer (PTC) is the most common thyroid cancer, which accounts for nearly 90% of all thyroid malignant tumors (2). Cervical lymph node metastases present at the time of diagnosis in 12%-81% of patients with PTCs (3). The spread of lymph node metastasis tends to progress from the central neck adjacent to thyroid to the lateral cervical compartments (4). Although controversy exists, most studies supported the prognostic significance of regional lymph node metastases in differentiated thyroid cancer (5–8). For patients with clinically apparent or biopsy-proven cervical lymph node metastasis (N1b), therapeutic neck dissection is routinely performed synchronously with thyroidectomy. For patients without metastasis to lateral neck, the role of routine prophylactic central neck dissection remains controversial. While some studies suggested that prophylactic dissection could improve disease-specific survival and local recurrence (9, 10), more other studies showed that prophylactic dissection had no improvement in locoregional recurrence but increased the likelihood of temporary morbidity (11–14). Before consensus has been reached, preoperative prediction of central cervical lymph node (CCLN) metastasis with adequate accuracy is valuable to avoid unnecessary central neck dissection in patients diagnosed as suspicious PTC or PTC in fine-needle aspiration (FNA).

Ultrasound (US) is the most important method to assess thyroid and cervical lymph nodes. However, the diagnostic efficiency of US in detecting CCLN metastasis is poor than that in lateral cervical lymph node (LCLN). A meta-analysis including 19 studies reported the pooled sensitivity, specificity, diagnostic odds ratio and area under curve of US in detecting CCLN metastasis to be 0.33, 0.93, 5.63, and 0.69, respectively; and LCLN metastasis to be 0.70, 0.84, 18.7 and 0.88, respectively (15). Nevertheless, US alone is not accurate enough for preoperative assessment of CCLN metastasis.

*BRAF* mutation is the most common genetic alteration in thyroid cancer, with incidence ranging from approximate 30% to more than 80% (16, 17). As one form of Raf kinases, BRAF is the most potent activator of the MAP kinase pathway, which plays an important role in tumorigenesis (18, 19). The diagnostic and prognostic value of *BRAF* mutation in thyroid cancer has been well documented (16). Recently, numerous studies investigated the association between *BRAF* mutation and (central) cervical lymph node metastasis in thyroid cancer. Some studies demonstrated *BRAF* mutation was correlated with (central) cervical lymph node metastasis (20, 21), while some others obtained the opposite conclusion (22–24). Therefore, this study aimed to investigate the association between CCLN metastasis and factors including clinical characteristics, US findings of thyroid and cervical lymph node, and *BRAF* mutation in PTC, and then develop a model to predict the presence of CCLN metastasis in FNA reporting suspicious PTC or PTC without lateral neck metastasis.

## Patients and Methods

### Patient Selection

**Figure 1:**
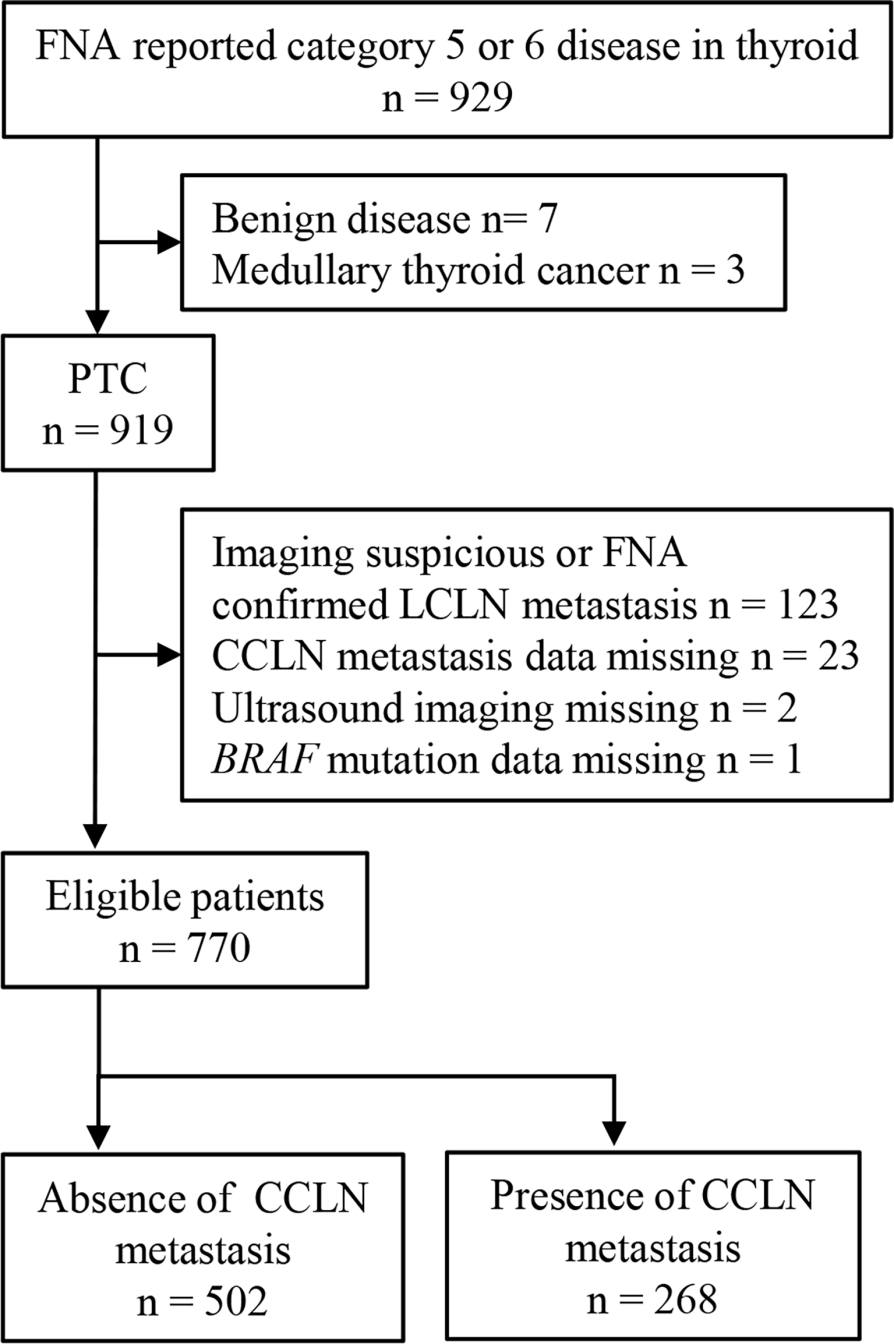
The flow chart of patient selection.

Consecutive inpatients who had US-guided FNA reporting suspicious PTC or PTC (25) and underwent partial or total thyroidectomy and CCLN dissection between May 1^st^, 2016 and June 30^th^, 2018 were retrieved. The institutional review board had approved this retrospective study. The retrieved patients were reviewed for eligibility. The inclusion criteria included: 1) histologically confirmed PTC, 2) detection of *BRAF* mutation for thyroid lesions with specimens from preoperative FNA, 3) underwent preoperative US examination of thyroid and neck. The exclusion criteria included: 1) imaging suspicious or FNA confirmed LCLN metastasis, 2) CCLN metastasis data was missing, 3) *BRAF* mutation data was missing, 4) US images were missing. The flow chart of patient selection was showed in **Figure 1**.

### US Examination of Thyroid and Neck

US examination of thyroid and neck was conducted with Logic E9 (GE Healthcare, Kretz, Zipf, Austria), IU22 (Philips Medical Systems, Bothell, WA), Aixplorer (Supersonic Imaging, Aix-en-Provence, France), Aplio 500 (Toshiba medical system, Japan), and Mylab90 (Esaote, Genoa, Italy) equipped with a 5–14-MHz linear-array transducer. First, the grey-scale images of the neck were obtained for thorough evaluation of the neck anatomy, including surrounding vasculature, major salivary glands, cervical lymph nodes, and thyroid. The cervical lymph node is divided into seven levels according to their anatomic location. The CCLNs refer to the level VI and VII, whereas the LCLNs include level I, II, III, IV, and V (26). Lymph node with any of following features was considered as positive for metastasis: hypoechoic, rounded, absence of fatty hilum, cystic or partially cystic, and/or microcalcifications. For thyroid, a suspected malignant nodule was carefully evaluated for the following features: 1) size, 2) location (upper 1/3, middle 1/3, lower 1/3, isthmus), 3) component (solid, cystic-solid), 4) echogenicity (hypoechoic, iso/hyperechoic), 5) margin (circumscribed, not circumscribed), 6) shape (round/oval, irregular), 7) ratio of tall to wide (less than 1, equal/more than 1), 8) calcification (no/macrocalcification, microcalcification), 9) capsule involvement (less than 1/4 involved, equal or more than 1/4 involved), 10) depth (dorsal, ventral), 11) multifocality (solitary, multiple in unilateral lobe, multiple in bilateral lobes). Calcification in a thyroid nodule with size equal or less than 1.5 mm was defined as microcalcification, otherwise macrocalcification. Multiple nodules were defined as more than one solitary suspected malignant nodules in thyroid. In the setting of multiple nodules, the US feature of thyroid nodule was extracted from the most suspicious one, which was indicated by the malignant radiologic features or growth pattern over time.

### US-Guided FNA Biopsy

The suspicious nodule in thyroid and neck was directed to US-guided FNA biopsy. Standard 21- or 23- gauge injection needles were used for FNA biopsy. Three to four passes per target nodule were made at the discretion of operator; two passes for cytologic diagnosis and one or two passes for BRAF mutation analysis. For both unilateral and bilateral multiple nodules in the thyroid, only the most suspicious was directed as target nodule. Specimens for cytologic diagnosis were evaluated by pathologist special for cytopathology and the results were reported according to The Bethesda system (25).

### *BRAF* Mutation Analysis

*BRAF* V600E mutation analysis was conducted in the Pathology Department. Briefly, the DNA of specimens from FNA biopsy was extracted. Real-time PCR was performed using the BioRad-CFX96 real-time PCR system (Bio-Rad, Hercules, CA, USA). The mutant *BRAF* gene (encoding *BRAF* V600E) was amplified with specific primers. Thermal cycling conditions were initial denaturation of 1 cycle for 5 minutes at 95°C, followed by 15 cycles of 95°C for 25 s, 64°C for 20 s, and 72°C for 20 s with a final step of annealing and elongation of 31cycles at 93°C for 25 s, 60°C for 35 s and 72°C for 20 s. The *BRAF* V600E mutation status of each primary PTC was determined using the Human *BRAF* Gene V600E Mutation Fluorescence Polymerase Chain Reaction (PCR) Diagnostic Kit (Amoy Diagnostics). The FAM signals of the mutation detection system indicate the mutation status of the sample. The HEX/VIC signals indicate the internal control status. The FAM Ct value was checked for each sample: a) If the sample FAM Ct value ≥27, the sample was classified as negative or below the detection limit of the kit. b) If the sample FAM Ct value < 27, the sample was classified as mutation positive. In the end, the run files were interpreted according to the manufacturer’s instruction.

### Criteria for Thyroidectomy and Central Neck Dissection

Total thyroidectomy and bilateral central neck dissection were performed in patients with bilateral thyroid cancer. For patients with unilateral cancer, the extent of thyroid surgery and ipsilateral central neck dissection were decided at the discretion of operator before the operation.

### Statistical Analysis

The end-point event of this study was CCLN metastasis. For bilateral cancer, only the CCLNs ipsilateral to the FNA-targeted nodule was analyzed. The total group of patients was randomly divided into a training set and an internal validation set with the ratio of 7:3. The predicting model was developed using the training set. Univariate analysis with Chi-squared test was used to identify the variables correlated with CCLN metastasis. Factors significantly correlated with CCLN metastasis in the univariate analysis were included in the multivariate analysis, which was conducted with logistic regression analysis. Factors with *p* < 0.05 in the regression analysis were used to build the predicting model. The efficacy of this model was evaluated with area under the curve (AUC), sensitivity, specificity and accuracy and validated in the validation set. All tests were two-sided, and *p* < 0.05 indicated statistical significance. The SPSS version 19.0 (IBM Corp., Armonk, NY, USA) software was used for all statistical analysis.

## Results

### Patient Characteristics

A total of 770 patients, including 580 (75.3%) females and 190 (24.7%) males, with median age of 44 years (range 22 - 80 years) were eligible for this study. Among them, 251 (32.6%) patients were accompanied with Hashimoto’s thyroiditis. Five hundred and fifty-three (71.8%) patients had one solitary tumor, whereas unilateral multiple tumors were found in 71 (9.2%) patients, and bilateral multiple tumors in 146 (19.0%) patients. The median size of tumors was 8 mm (range 3 -53 mm). *BRAF* mutation presented in 642 (83.4%) tumors. Only 79 (10.3%) patients presented suspicious CCLN metastasis in US. Total thyroidectomy was performed in 192 (24.9%) patients, partial thyroidectomy in the remaining patients. All patients accepted central neck dissection. After surgery, 268 (34.8%) patients had histologically confirmed CCLN metastasis, while the remaining (65.2%) patients did not. The mean number of removed lymph nodes was 4.2 (range 1 to 30), and mean number of positive lymph nodes was 2.1 (range 1 to 11). Five hundred and forty-six patients (70.9% of 770) were allocated into the training set. The patient characteristics in the training set were in good agreement with that in the validation set except slightly difference in the tumor location (**Table 1**).

**Table 1:** Patient characteristics.

Factors		Total No.	Patient sets (%)		p
			Training	Validation	
Gender	Female	580	414 (75.8)	166 (74.1)	0.616
	Male	190	132 (24.2)	58 (25.9)	
Age	≤ 40 years	286	207 (37.9)	79 (35.3)	0.490
	> 40 years	484	339 (62.1)	145 (64.7)	
Hashimoto’s thyroiditis	No	519	369 (67.6)	150 (67.0)	0.868
	Yes	251	177 (32.4)	74 (33.0)	
*BRAF* mutation	No	128	91 (16.7)	37 (16.5)	0.960
	Yes	642	455 (83.3)	187 (83.5)	
Cytologic category	V	137	98 (17.9)	39 (17.4)	0.859
	VI	633	448 (82.1)	185 (82.6)	
Multifocality	Solitary	553	394 (72.2)	159 (71.0)	0.551
	Unilateral multiple	71	53 (9.7)	18 (8.0)	
	Bilateral multiple	146	99 (18.1)	47 (21.0)	
Size	< 10 mm	479	346 (63.4)	133 (59.4)	0.299
	≥ 10 mm	291	200 (36.6)	91 (40.6)	
Location	Upper	125	95 (17.4)	30 (13.4)	0.024
	Middle	490	343 (62.8)	147 (65.6)	
	Lower	138	101 (18.5)	37 (16.5)	
	Isthmus	17	7 (1.3)	10 (4.5)	
Component	Solid	763	541 (99.1)	222 (99.1)	0.976
	Cystic-solid	7	5 (0.9)	2 (0.9)	
Echogenicity	Hypoechoic	725	510 (93.4)	215 (96.0)	0.166
	Iso/hyperechoic	45	36 (6.6)	9 (4.0)	
Margin	Circumscribed	288	201 (36.8)	87 (38.8)	0.598
	Not circumscribed	482	345 (63.2)	137 (61.2)	
Shape	Round/oval	390	268 (49.1)	122 (54.5)	0.175
	Irregular	380	278 (50.9)	102 (45.5)	
Ratio of tall to wide	< 1	247	179 (32.8)	68 (30.3)	0.512
	≥ 1	523	367 (67.2)	156 (69.7)	
Calcification	No/macrocalcification	239	172 (31.5)	67 (29.9)	0.665
	Microcalcification	531	374 (68.5)	157 (70.1)	
Capsule involvement	< 1/4	554	395 (72.3)	159 (71.0)	0.702
	≥ 1/4	216	151 (27.7)	65 (29.0)	
Depth	Dorsal	154	446 (81.7)	170 (75.9)	0.068
	Ventral	616	100 (28.3)	54 (24.1)	
US CCLNM	Negative	691	492 (90.1)	199 (88.8)	0.598
	Positive	79	54 (9.9)	25 (11.2)	
Thyroidectomy	Total	192	134 (24.5)	58 (25.9)	0.694
	Partial	578	412 (75.5)	166 (74.1)	
CCLNM	Yes	268	190 (34.8)	78 (34.8)	0.995
	No	502	356 (65.2)	146 (65.2)	

CCLNM, central cervical lymph node metastasis.

### Univariate Analysis

The association between CCLN metastasis and 17 factors including gender, age, accompanied with Hashimoto’s thyroiditis or not, *BRAF* mutation status, cytologic category, multifocality, size, location, component, echogenicity, margin, shape, ratio of tall to wide, calcification, capsule involvement, depth, and US CCLN status was analyzed (**Table 2**). The results indicated that 10 factors including gender, age, *BRAF* mutation status, cytologic category, multifocality, size, ratio of tall to wide, calcification, capsule involvement, and US CCLN status were significantly associated with CCLN metastasis.

**Table 2:** Univariate analysis.

Factors		Total No.	Central CLN metastasis (%)		p
			No	Yes	
Gender	Female	414	284 (68.6)	130 (31.4)	0.003
	Male	132	72 (54.5)	60 (45.5)	
Age	≤ 40 years	207	117 (56.5)	90 (43.5)	0.001
	> 40 years	339	239 (70.5)	100 (29.5)	
Hashimoto’s thyroiditis	No	369	240 (65.0)	129 (35.0)	0.909
	Yes	177	116 (65.5)	61 (34.5)	
*BRAF* mutation	No	91	69 (75.8)	22 (24.2)	0.020
	Yes	455	287 (63.1)	168 (36.9)	
Cytologic category	V	98	73 (74.5)	25 (25.5)	0.033
	VI	448	283 (63.2)	165 (36.8)	
Multifocality	Solitary	394	273 (69.3)	121 (30.7)	0.002
	Unilateral multiple	53	33 (62.3)	20 (37.7)	
	Bilateral multiple	99	50 (50.5)	49 (49.5)	
Size	< 10 mm	346	265 (76.6)	81 (23.4)	< 0.001
	≥ 10 mm	200	91 (45.5)	109 (54.5)	
Location	Upper	95	65 (68.4)	30 (31.6)	0.547
	Middle	343	224 (65.3)	119 (34.7)	
	Lower	101	64 (63.4)	37 (36.6)	
	Isthmus	7	3 (42.9)	4 (57.1)	
Component	Solid	541	352 (65.1)	189 (34.9)	0.432
	Cystic-solid	5	4 (80.0)	1 (20.0)	
Echogenicity	Hypoechoic	510	330 (64.7)	180 (35.3)	0.360
	Iso/hyperechoic	36	26 (72.2)	10 (27.8)	
Margin	Circumscribed	201	136 (67.7)	65 (32.3)	0.357
	Not circumscribed	345	220 (63.8)	125 (36.2)	
Shape	Round/oval	268	183 (68.3)	85 (31.7)	0.138
	Irregular	278	173 (62.2)	105 (37.8)	
Ratio of tall to wide	< 1	179	99 (55.3)	80 (44.7)	0.001
	≥ 1	367	257 (70.0)	110 (30.0)	
Calcification	No/macrocalcification	172	130 (75.6)	42 (24.4)	0.001
	Microcalcification	374	226 (60.4)	148 (39.6)	
Capsule involvement	< 1/4	395	345 (87.3)	50 (12.7)	< 0.001
	≥ 1/4	151	11 (7.3)	140 (92.7)	
Depth	Dorsal	446	295 (66.1)	151 (33.9)	0.329
	Ventral	100	61 (61.0)	39 (39.0)	
US CCLNM	Negative	492	356 (72.4)	136 (27.6)	< 0.001
	Positive	54	0 (0)	54 (100)	

CCLNM, central cervical lymph node metastasis.

### Multivariate Analysis

Factors significantly correlated with CCLN metastasis in the univariate analysis were included in the multivariate analysis. The results showed that age, *BRAF* mutation status, multifocality, size, and capsule involvement were the independent predictor of CCLN metastasis (**Table 3**). However, the calculated 95%CI of odds ratio (OR) for US CCLN status ranges from 0 to ∞ due to absence of patient positive for US CCLN status but without pathological confirmed CCLN metastasis (**Table 2**). Besides, US is the most important way to evaluate cervical lymph nodes in clinical. Therefore, US CCLN status was still included in the predicting model.

**Table 3:** Multivariate analysis.

Factors	*β*	*OR (95%CI)*	*p*
Gender	0.549	1.732 (0.858-3.496)	0.125
Age	-0.701	0.496 (0.262-0.940)	0.032
*BRAF* mutation	1.040	2.828 (1.165-6.866)	0.022
Cytologic category	-0.365	0.694 (0.313-1.542)	0.370
Multifocality	–	–	0.001
Unilateral Multiple	0.834	2.301 (0.891-5.945)	0.085
Bilateral multiple	1.606	4.985 (2.073-11.986)	< 0.001
Size	0.865	2.376 (1.196-4.721)	0.013
Ratio of tall to wide	0.046	1.047 (0.512-2.140)	0.900
Calcification	0.404	1.497 (0.716-3.132)	0.284
Capsule involvement	4.393	80.917 (36.768-178.080)	< 0.001
US CCLNM	22.053	3.7E+09 (0-∞)	0.996
Constant	-3.536	0.029	< 0.001

CCLNM, central cervical lymph node metastasis.

### Model Construction and Evaluation of Its Efficiency

The independent predictors and US CCLN status were put into the logistic regression analysis to build a predicting model (**Table 4**). Thus, the following model was built:


logit(p)=−3.426−0.658x1+1.055x2+0.784x3+1.605x4+0.949x5+4.344x6+22.013x7

**Table 4:** Model based on the independent predictors.

Factors	*β*	*OR (95%CI)*	*p*
Age	-0.658	0.518 (0.276-0.972)	0.040
*BRAF* mutation	1.055	2.872 (1.205-6.849)	0.017
Multifocality	–	–	0.003
Unilateral Multiple	0.784	2.190 (0.863 -5.556)	0.099
Bilateral multiple	1.605	4.978 (2.106-11.766)	< 0.001
Size	0.949	2.584 (1.384-4.826)	0.003
Capsule involvement	4.344	77.007 (35.600-166.574)	< 0.001
US CCLNM	22.013	3.6E+09 (0-∞)	0.996
Constant	-3.426	0.033	< 0.001

in which, p denotes the probability of CCLN metastasis, x1 denotes age > 40 years, x2 denotes *BRAF* mutation, x3 denotes unilateral multiple tumors, x4 denotes bilateral multiple tumors, x5 denotes size ≥ 10 mm, x6 denotes capsule involvement, x7 denotes positive US CCLN.

**Figure 2:**
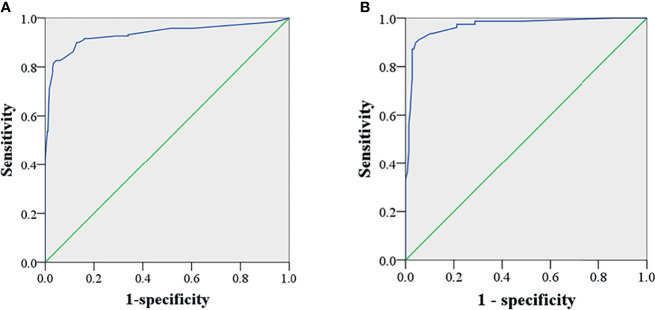
ROC of the test and validation set. The AUC was 0.933 (95%CI: 0.905-0.960, p < 0.001) and 0.967 (95%CI: 0.943-0.991, p < 0.001) in the training **(A)** and the internal validation set **(B)**, respectively.

The AUC of this model in predicting CCLN metastasis in the training set was 0.933 (95%CI: 0.905-0.960, p < 0.001). With the optimum cutoff of 0.465, the sensitivity, specificity and accuracy of this model were 0.816, 0.966 and 0.914, respectively. When validated in the internal validation set, the modal yielded the AUC of 0.967 (95%CI: 0.943-0.991, p < 0.001) (**Figure 2**). And the sensitivity, specificity and accuracy of this model in the validation set were 0.897, 0.959 and 0.936, respectively.

## Discussion

The supposed benefit of prophylactic CCLN dissection in thyroid cancer without metastasis to lateral neck includes local-recurrence prevention and improved long-term survival. However, although several meta-analyses showed reduction in the risk of local-recurrence in thyroid cancer with prophylactic central neck dissection, they all stated that the surgical morbidity should not be overlooked (27–29). Furthermore, a previous prospective study showed no outcome advantage but reduction in the rate of radioiodine treatment for prophylactic central neck dissection after 5-year’s follow-up (12). The evidence quality is low, so that prophylactic central neck dissection for advanced thyroid cancer such as T3 or T4 or cN1b disease was stated as weak recommendation in the latest management guidelines of American thyroid association for differentiated thyroid cancer published in 2016 (30). Therefore, it would be more rational to perform central neck dissection in selective thyroid cancer with high risk of CCLN metastasis.

Unfortunately, to date no non-invasive imaging method can confidently classify cervical lymphadenopathy as metastasis, especially for CCLNs because they may be obstructed by the thyroid. In this study, the sensitivity of US alone for detection of CCLN metastasis was only 29.5% (79/268), though the specificity was 100% (502/502), which are comparable with previous report (15). A recent meta-analysis demonstrated that computed tomography performed poorly as US did in identifying the presence of CCLN metastasis (31). In view of the low efficiency of unimodal imaging, we tried to develop a model incorporating factors of clinicopathology, imaging and gene mutation that could be determined preoperatively to predict the CCLN metastasis. Finally, a simple predicting model was successfully built and yielded AUC of 0.933 and 0.967 in the training set and validation set, respectively, indicating the excellent discrimination and robustness of this model.

Several studies have developed various models to predict the CCLN metastasis in thyroid cancer (32–37). In comparison with these models, there are several advantages in our study. Firstly, the outward spread mode of metastasis indicates that 1) patients with LCLN metastasis are very likely accompanied with CCLN metastasis, 2) patients without LCLN metastasis may have CCLN metastasis with unknown possibility. Published data showed that patients with concurrent LCLN and CCLN metastasis accounted more than 70% of patients (155/211, ref. 37) with LCLN metastasis. Consequently, patients with suspicious or confirmed LCLN metastasis almost always undergo neck dissection covering lateral and central cervical compartment in clinical. The controversy in clinical right now is that whether there is a need to undergo central neck dissection in patients without evidence of LCLN metastasis. Patients with suspicious or confirmed LCLN metastasis therefore were excluded in our study, but not in other previous studies, making the focus of our study more relevant to the clinical problem. Secondly, our model incorporated not only clinicopathological and imaging factors commonly included in previous studies, but also *BRAF* gene mutation. Thirdly, *BRAF* mutation status was determined with specimens from the preoperative FNA biopsy. Therefore, all factors included in this model could be determined preoperatively, making it a real tool to preoperatively predict the status of CCLN metastasis. Finally, this simple model showed more excellent discrimination in both the training set and internal validation set than most previous models.

One interesting finding in this study is that CCLN metastasis was less likely to present in older patients with PTC, which was similar to previous reports (37, 38). This indicates that thyroid cancer may be more inert in old patients than in young counterparts, although the molecular mechanism is unclear. However, the impact of lymph node metastasis on survival is more apparent in older patients, which is the reason for that the change of N stage changes the prognostic stage in old patients (with T1/M0 or T2/M0 disease), but not in young patients in the AJCC manual (26). In other words, lymph node metastasis occurs in a less probability in older patients; but once it occurs, it is more detrimental.

Consistent with previous studies (17, 37–39), factors such as larger size, multifocality and capsule involvement which indicate heavier tumor burden and invasiveness without out of expectation were independent predictors and included in the model. Besides, the results of this study also supported that *BRAF* mutation was significantly associated with CCLN metastasis in PTC. However, although gender and calcification were significantly correlated with CCLN metastasis in the univariate analysis, they did not show statistical significance in the multivariate analysis.

One confounder should be noted in this study is that only unilateral CCLN ipsilateral to the FNA-targeted nodule was analyzed in this study. However, for patients suffered from bilateral thyroid cancer, metastasis might come from contralateral disease; for patients with unilateral cancer, metastasis might skip to contralateral CCLNs. Although no study reports how often this would happen yet, we believe it occurs rarely and would result in little impact on the results. Other limitations of this study include lack of external validation and retrospective design.

In conclusion, this study demonstrated that age, *BRAF* mutation, multifocality, size, and capsule involvement were independent predictors of CCLN metastasis in FNA reporting suspicious PTC or PTC without lateral neck metastasis. A simple model was successfully built based on these factors and US CCLN status and shown excellent discrimination to distinguish FNA reporting suspicious PTC or PTC with or without CCLN metastasis.

## Data Availability Statement

The original contributions presented in the study are included in the article/supplementary material. Further inquiries can be directed to the corresponding author.

## Ethics Statement

The studies involving human participants were reviewed and approved by Ethics Committee of Fudan University Shanghai Cancer Center. Written informed consent for participation was not required for this study in accordance with the national legislation and the institutional requirements.

## Author Contributions

KZ, conception, writing and supervision. LQ, writing, data collection and analysis. JC, writing and data analysis. QZ and CC, data collection and review. All authors contributed to the article and approved the submitted version.

## Conflict of Interest

The authors declare that the research was conducted in the absence of any commercial or financial relationships that could be construed as a potential conflict of interest.

## Publisher’s Note

All claims expressed in this article are solely those of the authors and do not necessarily represent those of their affiliated organizations, or those of the publisher, the editors and the reviewers. Any product that may be evaluated in this article, or claim that may be made by its manufacturer, is not guaranteed or endorsed by the publisher.
